# Effects of a Mediterranean diet and structured exercise intervention on selected anthropometric, cardiovascular, and metabolic variables in physically inactive adults: a randomized controlled trial

**DOI:** 10.3389/fnut.2025.1695412

**Published:** 2025-11-12

**Authors:** Pablo Prieto-González, Fatma Hilal Yagin, Abdullah F. Alghannam, Umut Canli

**Affiliations:** 1 Sport Sciences and Diagnostics Research Group, GSD-HPE Department, Prince Sultan University, Riyadh, Saudi Arabia; 2 Department of Biostatistics, Faculty of Medicine, Malatya Turgut Ozal University, Battalgazi, Türkiye; 3 Department of Computer Science, Lakehead University, Thunder Bay, ON, Canada; 4 Lifestyle and Health Research Center, Health Sciences Research Center, Princess Nourah Bint Abdulrahman University, Riyadh, Saudi Arabia; 5 Faculty of Sports Sciences, Tekirdağ Namik Kemal University, Tekirdağ, Türkiye

**Keywords:** Mediterranean diet, cardiometabolic health, anthropometric variables, metabolic variables, cardiovascular variables, lifestyle intervention, structured exercise

## Abstract

**Objective:**

The study aims to determine whether the combined implementation of Mediterranean diet (MD) adherence and structured physical exercise contributes to improvements in body composition and cardiometabolic health indicators in a physically inactive but otherwise healthy adult population.

**Methods:**

A randomized controlled trial (RCTs) was conducted with 125 physically inactive adults (61 males, 64 females) aged 35–50 years, free from cardiovascular, metabolic, or musculoskeletal conditions. Participants were assigned to either an 8-week intervention group (*n* = 62: 30 males, 32 females) combining Mediterranean diet adherence and supervised combined training (three endurance and two resistance sessions per week) or a control group (*n* = 63: 31 males, 32 females) instructed to maintain habits. Anthropometric, cardiovascular, and metabolic variables were assessed pre- and post-intervention under standardized conditions. A 2 × 2 × 2 mixed-design ANOVA (group × sex × time) was conducted, with Tukey's *post hoc* tests applied when significant differences were found.

**Results:**

Significant differences over time, between sexes, and between groups were observed in anthropometric, cardiovascular, and metabolic variables. In the experimental group (EG), both men and women experienced significant reductions in body mass (BM), BMI, fat percentage, waist circumference, waist-to-hip ratio (WHR), systolic and diastolic blood pressure, heart rate, double product, glucose, and low-density lipoprotein (LDL) cholesterol from pre- to post-test (all *p* < 0.05, with effect sizes ranging from small to large). Lean mass increased significantly only in the EG, while high-density lipoprotein (HDL) levels improved predominantly in women. Men and women differed significantly in body mass, BMI, fat percentage, lean mass, waist circumference, waist-to-hip ratio, heart rate, double product, glucose, HDL, and triglycerides at both time points (all *p* < 0.001). The EG showed significantly greater improvements compared to the control group after the intervention (*p* < 0.001 for most variables), confirming the intervention's effectiveness.

**Conclusion:**

This study provides robust evidence that a lifestyle intervention combining Mediterranean diet adherence with structured physical exercise is an effective and feasible strategy to enhance cardiometabolic health in physically inactive adults. Its multicomponent nature and consistent benefits across sexes support its integration into preventive health programs. Further research is warranted to assess the sustainability of these outcomes and their generalizability to broader populations.

## 1 Introduction

Health plays a pivotal role in overall wellbeing and is strongly influenced by lifestyle habits, particularly diet and physical activity (1). Among dietary patterns, the Mediterranean diet (MD) stands out for its well-documented benefits in cardiovascular health, weight management, and chronic disease prevention. Rich in plant-based foods, healthy fats such as olive oil, and moderate amounts of animal-based protein, the MD promotes overall health and helps prevent multiple illnesses (2).

The MD has been the subject of scientific investigation since the 1960s and is considered one of the most extensively studied dietary patterns worldwide. In 2010, UNESCO recognized it as an Intangible Cultural Heritage of Humanity, emphasizing not only its health-promoting properties but also its social, cultural, and environmental dimensions (3). Central to this model are shared meals, traditional farming methods, and an inherent link to physical activity, all contributing to ecological sustainability and biodiversity preservation. Its origins can be traced to countries such as Spain, Italy, and Greece, shaped by the region's climate, geography, and cultural traditions (4).

The MD emphasizes frequent consumption of seasonal plant-based foods—fruits, vegetables, nuts, and whole grains—with olive oil as the primary fat source. Fish, dairy, poultry, and eggs are consumed in moderation, whereas red and processed meats are eaten infrequently. Its nutrient profile, rich in monounsaturated and polyunsaturated fats, omega-3 fatty acids, fiber, natural antioxidants, and phytoactive compounds, together with low levels of saturated and trans fats, provides essential minerals, vitamins, and proteins (5). These properties are linked to preventive effects against cancer, type 2 diabetes, cardiovascular diseases, metabolic syndrome, obesity, hypertension, and Alzheimer's disease, as well as improvements in cardiovascular symptoms, weight reduction, and increased life expectancy (6).

Recent evidence suggests that these health benefits are amplified when the MD is combined with regular physical activity, particularly in populations with obesity or metabolic syndrome (7). Physical activity—aligned with World Health Organization (WHO) recommendations—is associated with improvements in cardiovascular health, body composition, mental health, and quality of life, and plays a key role in the prevention and management of non-communicable diseases such as type 2 diabetes, cardiovascular disease, stroke, and certain cancers (8, 9). The WHO advises that adults aged 18–64 engage in at least 150 min of moderate-intensity activity or 75 min of vigorous-intensity activity weekly, or an equivalent combination, along with muscle-strengthening activities involving major muscle groups at least twice per week (9, 10).

Despite these benefits, global data reveal concerning trends: one-third of adults and 81% of adolescents fail to meet recommended activity levels. This problem, exacerbated by economic development, transportation changes, technological dependence, and increasingly sedentary lifestyles, contributes to rising rates of obesity, cardiovascular disease, and mental health disorders (9, 11). Physical inactivity accounts for an estimated 4–5 million preventable deaths annually, disproportionately affecting women, older adults, and individuals with disabilities. Adults aged 35–50 years represent a critical demographic window for preventive interventions, as this age range coincides with the onset of age-related metabolic decline and increased cardiovascular risk factors, yet precedes the development of established chronic diseases that may limit intervention effectiveness (12). Research indicates that lifestyle interventions in this age group show optimal responsiveness to combined diet–exercise protocols, with greater potential for long-term behavior modification compared to older populations (13). The WHO projects that without improvement in physical activity levels, nearly 500 million new cases of preventable non-communicable diseases will occur between 2020 and 2030, costing $300–520 billion in healthcare expenditures, with annual global costs reaching $47.6 billion (9, 14).

The rationale for a synergistic effect between MD adherence and structured exercise lies in their complementary mechanisms of action: the MD provides anti-inflammatory compounds, antioxidants, and an optimal macronutrient profile supporting metabolic health, while physical activity enhances insulin sensitivity, promotes fat oxidation, preserves lean mass, and improves cardiovascular function. Together, they may address multiple pathways simultaneously, yielding greater health benefits than either alone. Emerging evidence supports this synergy, particularly for improving insulin resistance, lipid profiles, and cardiovascular risk factors. However, much of the literature focuses on specific populations, such as individuals with obesity or diabetes (15, 16), and randomized controlled trials (RCTs) specifically combining MD with structured exercise remain scarce. Some studies have examined physical activity alongside hypocaloric diets (17, 18) or explored effects on body composition without focusing on the MD (19). Others report greater improvements in metabolic, cardiovascular, and anthropometric parameters from combining MD with exercise compared to single interventions, yet high-quality experimental evidence is still limited (20–22). Systematic reviews examining this combination reveal significant heterogeneity in study designs, population characteristics, and outcome measures, with fewer than five randomized controlled trials specifically addressing combined Mediterranean diet and exercise interventions in healthy adults (23).

Previous intervention studies combining dietary modifications with exercise have demonstrated significant cardiometabolic improvements within 6–12 weeks. However, systematic reviews reveal considerable heterogeneity in intervention protocols and conflicting results regarding optimal duration, with some studies showing plateauing effects after 6 weeks while others report continued improvements up to 16 weeks. Specifically, meta-analyses indicate that structured exercise interventions can yield measurable effects on body composition and cardiovascular parameters within 8–10 weeks (24), while Mediterranean diet adherence demonstrates rapid improvements in lipid profiles and inflammatory markers within 6–8 weeks of implementation (25). An 8-week timeframe may therefore be sufficient to observe meaningful physiological adaptations while maintaining participant compliance and minimizing dropout rates, which are commonly higher in longer lifestyle interventions.

Given this evidence base and the identified research gaps, observational research has shown that concurrent adherence to MD and physical activity significantly reduces all-cause mortality, but this does not substitute for evidence from controlled interventions (26). This gap highlights the need for experimental studies assessing the effects of combining MD adherence with structured exercise programs. While previous RCTs have demonstrated benefits of Mediterranean diet and exercise interventions in populations with existing metabolic disorders, several gaps remain. First, most studies have focused on individuals with established metabolic dysfunction rather than physically inactive but otherwise healthy adults as a preventive approach. Second, limited evidence exists on the implementation of culturally adapted Mediterranean diet interventions in non-Mediterranean populations, such as Saudi Arabia. Third, few studies have employed sex-stratified designs with a comprehensive analysis of sex-specific responses across multiple health domains.

Therefore, this study aimed to evaluate the effects of an intervention combining the Mediterranean diet and structured physical activity—based on WHO recommendations—on anthropometric, cardiovascular, and metabolic parameters in physically inactive adults. The findings are intended to provide robust evidence supporting integrated diet and exercise strategies as effective public health interventions.

## 2 Methods

### 2.1 Study design

A randomized controlled trial was conducted in accordance with the CONSORT guidelines.

### 2.2 Study participants

A total of 140 participants (70 males and 70 females), aged between 35 and 50 years and residing in Riyadh, were initially recruited through social media platforms and community health centers. Eligibility screening ensured that participants did not present any cardiovascular, respiratory, or metabolic conditions, nor any musculoskeletal injuries that could interfere with physical activity. Additionally, none of the participants engaged in regular or structured physical exercise, followed a prescribed diet, or were active smokers. Physical inactivity was confirmed using the short form of the International Physical Activity Questionnaire (IPAQ) (27), which evaluates whether participants met the World Health Organization's recommendations for both aerobic physical activity and muscle-strengthening exercises. Participants were classified as physically inactive if they engaged in <150 min per week of moderate-to-vigorous physical activity and <2 sessions per week of muscle-strengthening activities, below the WHO recommended thresholds (27).

Following initial screening, 15 individuals were excluded due to smoking, insulin-dependent diabetes, moderate-to-severe asthma, or neuromuscular injuries incompatible with training. Therefore, 125 participants (61 males and 64 females) completed the study and were included in the final analysis: 30 males in the experimental group (EG), 31 males in the control group (CG), 32 females in the EG, and 32 females in the CG.

All participants provided written informed consent prior to enrollment. The study was approved by the Institutional Review Board (IRB) of Prince Sultan University (PSU) under the Approval Letter of Authorization on Research Ethics (PSU IRB-2022-11-0133). All procedures were conducted in accordance with the ethical standards of the institutional committee and the principles outlined in the Declaration of Helsinki.

### 2.3 Interventions

The EG followed an 8-week combined training program consisting of two weekly resistance training sessions and three weekly aerobic endurance sessions. The intervention was designed in accordance with the World Health Organization's recommendations for physical activity in adults (9) and was supervised by a Doctor in Sports Sciences to ensure the proper application of fundamental training principles, such as progression, overload, individualization, and recovery (28). During the week preceding the implementation of the training protocol, all participants assigned to the EG underwent a familiarization phase (week 0), which consisted of both resistance and endurance training sessions performed twice a week, as outlined in Table 1.

**Table 1:** Weekly structure of the periodized resistance and endurance training program.

**Training modality**	**Resistance training**			**Endurance training**		
**Week**	**Sets**	**Repetitions**	**Rest**	**Duration per session**	**Total weekly time**	**Intensity (% HRmax)**
0	1	14	1'−2'	30'	60'	60%
1	3	14	1'−2'	40'	120'	64%−66%
2	4	14	1'−2'	50'	150'	64%−66%
3	5	14	1'−2'	55'	165'	66%−68%
4	5	14/14/12/12/10	1'−2'	60'	180'	68%−70%
5	5	14/12/10/10/10	1'−2'	55'	165'	70%−73%
6	4	10	1'−2'	50'	150'	73%−76%
7	4	10/8/10/8	1'−2'	45'	135'	77%−80%
8	3	8	1'−2'	40'	120'	80%−82%

Both components—resistance and endurance—followed a periodized structure. In the endurance training program (see Table 1), session volume (duration) was progressively increased during the initial weeks to promote physiological adaptation. In the latter weeks, intensity was gradually increased while volume was slightly reduced, ensuring a safe and effective progression (28). Endurance training sessions were performed on non-consecutive days (e.g., Sunday, Tuesday, and Thursday) to allow for adequate recovery. The structure and dose-response of the program were based on current public health recommendations for aerobic exercise in adults (29, 30). The endurance modality consisted of treadmill running, and training intensity was monitored during each session using heart rate monitors (Polar H10, Polar Electro Oy, Kempele, Finland) to ensure participants remained within their prescribed heart rate zones, calculated as a percentage of their estimated HRmax.

The resistance training program was likewise periodized, incorporating systematic variations in sets, repetitions, and rest intervals to promote muscular adaptation and avoid performance plateaus. Participants trained on non-consecutive days (e.g., Monday and Wednesday) using eight exercises that targeted all major muscle groups (see Table 1). During the first weeks, volume was progressively increased by adding sets, followed by a reduction in repetitions to increase intensity in the second half of the intervention. This structure aligned with evidence-based guidelines for strength development in both healthy adults and older (31, 32). Each session consisted of 3–5 sets per exercise, with repetition ranges between 8 and 15 and rest intervals between 60 s and 2 min, depending on the exercise type and intensity. Both free-weight and machine-based exercises were used to accommodate varying ability levels. Exercises targeting larger muscle groups were performed before those targeting smaller muscles, and multi-joint exercises were completed before single-joint movements, adhering to established strength training principles (31).

To individualize training intensity, a Repetitions in Reserve (RIR)-based prescription method was used. This approach allowed participants to self-regulate load by adjusting it based on the number of repetitions they believed they could still perform before reaching muscular failure. This strategy was a safer and more flexible alternative to percentage-based prescription methods, especially for populations with diverse physical capacities (32). It also enhanced adaptability and reduced fatigue accumulation, which is particularly relevant for untrained adults. A moderate RIR threshold (e.g., 2–3 RIR) was applied, in line with evidence from Mangine et al. (33), who reported that submaximal training with RIR preserved performance and training volume while minimizing perceived exertion and risk of overtraining (33).

Each training session began with a standardized warm-up comprising three phases. The first included 5 min of general activation with jogging, multidirectional movements, and dynamic drills (e.g., butt kicks, carioca, and lateral steps with hops). The second phase focused on joint mobility exercises performed in a cephalocaudal sequence for about 5 min. Finally, session-specific preparation was conducted: resistance sessions started with one light set (RIR ≥ 6) of selected exercises, while endurance sessions included 5 min of treadmill running at 60% HRmax to progressively raise heart rate and neuromuscular readiness.

Participants were required to attend at least 90% of all training sessions to be included in the final analysis. To promote adherence, participants received verbal reminders and motivational support from the research team during weekly supervised sessions throughout the intervention. All sessions were conducted at the same gym facility to control environmental variables and ensure protocol consistency. The same investigator supervised every session to monitor compliance, provide guidance, and ensure participant safety. Participants were also encouraged to communicate any difficulties or concerns promptly. The CG was instructed to maintain their usual lifestyle habits and avoid initiating any new structured exercise or dietary changes during the study period.

### 2.4 Dietary Intervention

Prior to the intervention, participants received structured education on the core principles of the Mediterranean Diet (MD), which promotes high consumption of plant-based foods, extra-virgin olive oil as the main fat source, moderate intake of fish, poultry, and dairy, and limited red and processed meat consumption (2). The MD is also embedded within a broader Mediterranean lifestyle that encourages sustainability, regular physical activity, and the social enjoyment of meals (34).

The MD plan included a structured five-meal daily format (breakfast, mid-morning snack, lunch, afternoon snack, and dinner), with each meal emphasizing plant-based and minimally processed ingredients. Meal composition was individualized to ensure nutritional adequacy and compliance with MD principles. An example of the prescribed meal plan for participants is provided in Table 2 below.

**Table 2:** Example of a daily mediterranean diet meal plan.

**Meal**	**Menu**
Breakfast	Whole-grain toast with fresh tomato and extra virgin olive oil, a handful of almonds, and one piece of seasonal fruit
Mid-morning snack	Natural yogurt with walnuts
Lunch	Lentil and vegetable stew, a side of mixed greens dressed with olive oil and vinegar, and a slice of whole-grain bread
Afternoon snack	Fresh fruit and a few olives
Dinner	Grilled sardines with steamed broccoli and brown rice, drizzled with olive oil and lemon

Adherence to the Mediterranean diet was assessed at baseline (week 0) and throughout the 8-week intervention (weeks 2, 4, 6, and 8) using the culturally adapted 13-item Arabic Mediterranean Diet Scale, which had been previously validated in Saudi populations (35). The MDS scores range from 0 to 13 and are categorized as low (≤ 5), moderate (6–9), or high (≥10), allowing for the evaluation of adherence levels despite cultural restrictions on alcohol consumption. MDS was analyzed as a continuous variable to track adherence changes over time, with participants maintaining mean scores ≥10 throughout the intervention period. The CG members were instructed to maintain their usual dietary habits. The results are presented in Table 3.

**Table 3:** Mediterranean diet adherence scores over time (Mean ± SD).

**Week**	**EG male**	**CG male**	**EG female**	**CG female**
0	7.09 ± 1.94	6.87 ± 1.79	7.17 ± 1.86	7.13 ± 1.89
2	10.58 ± 1.17	6.90 ± 1.62	10.92 ± 1.57	7.02 ± 1.82
4	10.89 ± 1.13	6.96 ± 1.67	11.15 ± 1.23	6.99 ± 1.93
6	11.13 ± 1.19	7.01 ± 1.64	11.32 ± 1.28	7.05 ± 1.87
8	11.25 ± 1.11	6.98 ± 1.61	11.27 ± 1.21	7.11 ± 1.88

Scores on the Mediterranean Diet Scale were categorized to reflect different levels of adherence: a score of 5 or below (≤ 5) indicated low adherence, scores between 6 and 9 reflected moderate adherence, and scores of 10 or higher (≥10) represented high adherence to the Mediterranean dietary pattern.

### 2.5 Outcomes

Anthropometric, cardiovascular, and metabolic variables were assessed at baseline and post-intervention by the same trained research team under standardized laboratory conditions. Assessments were performed between 8:00 a.m. and 10:00 a.m., following an overnight fast of at least 8 h and after a 48-h rest period to minimize acute training effects. Participants were instructed to abstain from caffeine and energy drinks for at least 48 h prior to testing. Study participants were encouraged to perform to the best of their capacity during all assessments, with verbal encouragement provided to ensure maximal effort and optimal performance outcomes (36, 37).

Anthropometric, cardiovascular, and metabolic variables were assessed at baseline and post-intervention by the same trained research team in a temperature-controlled room (20–24 °C). Anthropometric evaluations were performed with participants barefoot, wearing light clothing, and measurements were taken always on the right side of the body, in accordance with ISAK guidelines (65). Each anthropometric and cardiovascular parameter was measured three times, and the median value was recorded for each measurement. All equipment was previously calibrated using a sample of 50 individuals to ensure measurement accuracy.

#### 2.5.1 Anthropometric variables

Height (HE), body mass (BM), and body mass index (BMI) were measured using a digital column scale and stadiometer (Seca 769, Hamburg, Germany) with a precision of 0.1 kg and 0.1 cm, respectively. Body fat percentage (%FAT) was estimated via the sum of six skinfolds (abdominal, subscapular, triceps, supraspinal, quadriceps, and calf) using a Harpenden skinfold caliper (Model FG1056, Sussex, UK). The following validated equation was used to estimate the %FAT (38):

*%FAT* = [(Σ* of abdominal, supraspinal, subscapular, triceps*, *quadriceps, and calf skinfolds*) × 0.143] + 4.56

To measure the Fat-Free Mass (FFM), the following equation was used (39):

*FFM* = *Total weight* (*kg*) − *Fat mass* (*kg*)

Waist circumference (WAIST) was measured at the midpoint between the lower margin of the last palpable rib and the top of the iliac crest, using a flexible, non-elastic anthropometric tape. Hip circumference was measured at the widest portion of the buttocks, with participants standing upright, feet together, arms relaxed at the sides, and measurements taken at the end of a normal expiration. Each measurement was taken three times, and the median value was recorded for analysis. Waist-to-hip ratio (WHR) was calculated as waist circumference divided by hip circumference in accordance with WHO guidelines (40).

#### 2.5.2 Cardiovascular variables

Systolic and diastolic blood pressure (BP_SYS and BP_DIA), heart rate (BPM), and double product (DP) were assessed with participants seated and relaxed, following standardized procedures recommended by major clinical guidelines from the American Heart Association (AHA) and the American College of Cardiology (ACC) (41). Measurements were obtained using a validated Omron HEM-907XL Professional Digital Blood Pressure Monitor. Three consecutive readings were taken on the upper left arm at 1-min intervals. The median value of the three measurements for SBP, DBP, and BPM was recorded for analysis. Double product (DP) was calculated as SBP × BPM to estimate myocardial workload (42).

#### 2.5.3 Metabolic variables

Metabolic variables—including blood glucose (GLU), high-density lipoprotein (HDL), low-density lipoprotein (LDL), total cholesterol (CHO), triglycerides (TRYG), and uric acid (URI_AC)—were analyzed from venous blood samples collected under fasting conditions and processed in a certified clinical laboratory using standardized biochemical assays.

### 2.6 Sample size calculation

The required sample size was calculated using G^*^Power software based on an expected moderate effect size (*f* = 0.25), a significance level of 0.05, and a power of 0.80 for the primary outcome variables. The calculation indicated a minimum of 120 participants, considering a potential dropout rate of 10%−15%. Accordingly, 140 participants were recruited to ensure sufficient statistical power.

### 2.7 Randomisation

Block randomisation was performed separately by sex to ensure balanced groups for males and females. Participants were first ranked from highest to lowest BMI to create blocks of similar BMI values, given the strong correlation between BMI and health-related variables.

Within each block, participants were randomly assigned to the experimental (EG) or control group (CG) using a computerized random number generator, ensuring an unpredictable and unbiased allocation. This method guarantees that the distribution of participants across groups is balanced regarding both sex and BMI.

### 2.8 Statistical analysis

The results are presented as mean (M) ± standard deviation (SD). The normality of the data was assessed using the Kolmogorov–Smirnov test, and Levene's test was used to verify the homogeneity of variances. The Intraclass Correlation Coefficient (ICC) was calculated to assess the reliability of the anthropometric and health measurements. After confirming the assumptions of normality and homoscedasticity, a 2 × 2 × 2 mixed-design Analysis of Variance (ANOVA) was conducted to examine the effects of the Mediterranean diet and physical activity intervention, considering group (control vs. experimental), sex (male vs. female), and time (pre-test vs. post-test) as factors. This analysis allowed for the evaluation of main effects and interaction effects. When statistically significant differences were identified, Tukey's *post hoc* test was applied to determine specific group differences. The magnitude of the observed effects was reported using partial eta squared (η^2^), with values of 0.01, 0.06, and 0.14 representing small, medium, and large effect sizes, respectively (43). All statistical analyses were performed using IBM SPSS V.26^®^ software, with a significance level set at *p* < 0.05.

## 3 Results

All participants in the EG met the 90% attendance requirement and were included in the final analysis (*n* = 62; 30 males, 32 females). All CG participants completed the assessment sessions (*n* = 63; 31 males, 32 females). After verifying normality and homoscedasticity assumptions, ICC values showed high test–retest reliability across all study variables (see Table 4), most exceeding 0.90, indicating excellent measurement consistency. Body mass, body fat percentage, and blood glucose demonstrated particularly strong reliability in both sexes and groups, whereas waist circumference and triglycerides showed slightly lower but still acceptable ICC values (>0.85). Overall, these results confirmed the stability and robustness of the study measures.

**Table 4:** Intraclass correlation coefficient between pre-test and post-test of study variables, analyzed group (experimental and control), and sex (male and female).

**Variables/Group/Sex**	**ICC**	**Variables/Group/Sex**	**ICC**
BM_EG_MALE	0.985	BPM_EG_MALE	0.994
BM_EG_FEMALE	0.898	BPM_EG_FEMALE	0.974
BM_CG_MALE	0.994	BPM_CG_MALE	0.984
BM_CG_FEMALE	0.944	BPM_CG_FEMALE	0.911
BMI_EG_MALE	0.970	DP_EG_MALE	0.945
BMI_EG_FEMALE	0.954	DP_EG_FEMALE	0.970
BMI_CG_MALE	0.997	DP_CG_MALE	0.921
BMI_CG_FEMALE	0.976	DP_CG_FEMALE	0.899
%FAT_EG_MALE	0.986	GLU_EG_MALE	0.996
%FAT_EG_FEMALE	0.992	GLU_EG_FEMALE	0.987
%FAT_CG_MALE	0.961	GLU_CG_MALE	0.943
%FAT_CG_FEMALE	0.994	GLU_CG_FEMALE	0.904
LM_EG_MALE	0.989	HDL_EG_MALE	0.989
LM_EG_FEMALE	0.998	HDL_EG_FEMALE	0.987
LM_CG_MALE	0.991	HDL_CG_MALE	0.917
LM_CG_FEMALE	0.933	HDL_CG_FEMALE	0.895
WAIST_EG_MALE	0.973	LDL_EG_MALE	0.994
WAIST_EG_FEMALE	0.899	LDL_EG_FEMALE	0.993
WAIST_CG_MALE	0.905	LDL_CG_MALE	0.962
WAIST_CG_FEMALE	0.926	LDL_CG_FEMALE	0.986
WHR_EG_MALE	0.927	CHO_EG_MALE	0.997
WHR_EG_FEMALE	0.902	CHO_EG_FEMALE	0.992
WHR_CG_MALE	0.958	CHO_CG_MALE	0.976
WHR_CG_FEMALE	0.904	CHO_CG_FEMALE	0.984
BP_SYS_EG_MALE	0.902	TRYG_EG_MALE	0.959
BP_SYS_EG_FEMALE	0.913	TRYG_EG_FEMALE	0.895
BP_SYS_CG_MALE	0.917	TRYG_CG_MALE	0.919
BP_SYS_CG_FEMALE	0.897	TRYG_CG_FEMALE	0.927
BP_DIA_EG_MALE	0.996	URI_AC_EG_MALE	0.983
BP_DIA_EG_FEMALE	0.984	URI_AC_EG_FEMALE	0.913
BP_DIA_CG_MALE	0.901	URI_AC_CG_MALE	0.911
BP_DIA_CG_FEMALE	0.925	URI_AC_CG_FEMALE	0.924

BM, body mass; BMI, body mass index; %FAT, body fat percentage; FFM, fat-free mass; WAIST, waist circumference; WHR, waist-to-hip ratio; BP_SYS, systolic blood pressure; BP_DIA, diastolic blood pressure; BPM, beats per minute; DP, double product; GLU, blood glucose; HDL, high-density lipoprotein; LDL, low-density lipoprotein; CHO, total cholesterol; TRYG, triglycerides; URI_AC, uric acid; EG, experimental group; CG, control group; MALE, male participants; FEMALE, female participants; ICC, intraclass correlation coefficient.

### 3.1 Anthropometric outcomes

As shown in Table 5, the 2 × 2 × 2 ANOVA for body mass (BM) revealed significant main effects of time (*F*(1,121) = 15.32, *p* < 0.001, η^2^ = 0.13), sex (*F*(1,121) = 20.45, *p* < 0.001, η^2^ = 0.08), and group (*F*(1,121) = 18.77, *p* < 0.001, η^2^ = 0.09). Significant interactions were found for time × group, sex × group, and time × sex × group, while time × sex was not significant. *Post hoc* analyses (Tukey) showed significant pre–post improvements within the EG for both men (*p* = 0.043) and women (*p* = 0.002), as well as sex differences within both groups at pre- and post-test (*p* < 0.001 for all).

**Table 5:** Effects of mediterranean diet and physical activity intervention on anthropometric, cardiovascular, and metabolic variables: 2 × 2 × 2 Mixed ANOVA (Group × Sex × Time).

**Variable**	**Male**				**Female**			
	**Pre-test**		**Post-test**		**Pre-test**		**Post-test**	
	**EG**	**CG**	**EG**	**CG**	**EG**	**CG**	**EG**	**CG**
BM	77.99 ± 10.18	78.31 ± 11.03	76.11 ± 9.23^*^	78.24 ± 11.94	58.10 ± 10.40	58.60 ± 11.41	56.27 ± 10.17^*^	58.77 ± 11.48
HE	1.77 ± 0.12	1.77 ± 0.11	1.77 ± 0.12	1.77 ± 0.11	1.70 ± 0.09	1.71 ± 0.10	1.70 ± 0.09	1.71 ± 0.10
BMI	24.95 ± 3.91	24.29 ± 3.89	24.33 ± 3.78^*^	24.81 ± 3.75	20.29 ± 4.56	20.22 ± 4.53	19.23 ± 4.42^*^	20.27 ± 4.49
FAT%	19.33 ± 6.35	19.49 ± 6.45	18.24 ± 6.27^*^	19.68 ± 6.48	25.77 ± 5.93	25.98 ± 5.98	24.64 ± 5.66^*^	26.06 ± 5.99
LEAN	62.93 ± 10.46	62.97 ± 10.34	63.24 ± 10.60	62.99 ± 10.32	43.02 ± 8.42	44.22 ± 8.49	43.17 ± 8.37	43.92 ± 8.99
WAIST	89.18 ± 10.88	89.38 ± 10.89	86.99 ± 10.01^*^	89.58 ± 10.08	70.44 ± 8.48	70.55 ± 8.24	66.89 ± 8.28^*^	70.61 ± 8.20
WHR	0.91 ± 0.08	0.92 ± 0.07	0.89 ± 0.08^*^	0.91 ± 0.07	0.85 ± 0.07	0.84 ± 0.08	0.81 ± 0.07^*^	0.84 ± 0.08
BP_SYS	121.33 ± 15.22	121.66 ± 15.37	116.11 ± 14.94^*#^	121.88 ± 15.22	120.36 ± 15.11	121.31 ± 16.38	117.13 ± 15.61^*#^	120.55 ± 16.28
BP_DIA	81.33 ± 10.22	81.26 ± 10.05	78.73 ± 10.03^*#^	81.13 ± 9.98	81.60 ± 10.03	79.93 ± 9.61	77.66 ± 10.92^*#^	80.10 ± 10.23
BPM	63.33 ± 12.05	63.50 ± 11.96	58.43 ± 11.90^*#^	62.27 ± 12.23	66.76 ± 12.70	66.30 ± 13.95	64.20 ± 13.40^*#^	68.34 ± 13.11
DP	7,693.06 ± 1,231.53	7,739.90 ± 1,192.23	6,659.40 ± 1,202.97^*#^	7,719.63 ± 1,281.27	8,035.13 ± 1,364.69	8,256.83 ± 1,307.45	7,770.90 ± 1,330.63^*^	8,439.16 ± 1,312.97
GLU	92.96 ± 11.38	93.24 ± 12.28	87.48 ± 11.08^*#^	92.93 ± 12.33	89.03 ± 10.66	88.9 ± 11.51	86.93 ± 10.42^*#^	89.62 ± 11.68
HDL	48.63 ± 9.66	49.19 ± 10.98	49.91 ± 9.44^*^	50.42 ± 10.08	52.92 ± 10.95	53.92 ± 10.23	54.30 ± 10.41^*#^	52.95 ± 10.81
LDL	120.42 ± 21.84	121.15 ± 20.18	117.52 ± 20.72^*#^	121.46 ± 19.26	117.01 ± 18.57	116.97 ± 19.61	113.72 ± 18.64^*#^	117.73 ± 20.89
CHO	186.63 ± 30.25	187.12 ± 28.69	180.48 ± 29.05^*#^	188.65 ± 30.45	179.29 ± 27.69	180.24 ± 28.34	171.61 ± 29.71^*#^	180.47 ± 29.64
TRYG	86.19 ± 14.14	85.03 ± 13.44	82.23 ± 12.86^*#^	85.41 ± 13.67	77.75 ± 13.21	77.94 ± 12.36	70.37 ± 12.16^*#^	78.72 ± 12.69
URIC_AC	5.62 ± 0.91	5.73 ± 0.98	4.93 ± 0.90^*#^	5.66 ± 0.93	5.22 ± 0.80	5.19 ± 0.85	4.69 ± 0.89^*#^	5.26 ± 0.84

BM, body mass; BMI, body mass index; %FAT, body fat percentage; LEAN, lean mass; WAIST, waist circumference; WHR, waist-to-hip ratio; BP_SYS, systolic blood pressure; BP_DIA, diastolic blood pressure; BPM, beats per minute; DP, double product; GLU, blood glucose; HDL, high-density lipoprotein; LDL, low-density lipoprotein; CHO, total cholesterol; TRYG, triglycerides; URI_AC, uric acid; EG, experimental group; CG, control group; MALE, male participants; FEMALE, female participants.

^*^Significant difference between pre- and post-test (p <0.05).

^#^Significant difference between groups at post-test (EG vs. CG).

For BMI, main effects were significant for sex (*F*(1,121) = 17.55, *p* < 0.001, η^2^ = 0.08), time (*F*(1,121) = 22.14, *p* < 0.001, η^2^ = 0.11), and group (*F*(1,121) = 18.21, *p* < 0.001, η^2^ = 0.09). Significant interactions were observed for time × sex, sex × group, time × group, and time × sex × group. *Post hoc* results indicated significant pre–post improvements in the EG for both sexes (*p* < 0.001) and consistent sex differences in both groups at both time points (*p* < 0.001).

For FAT%, significant main effects were found for sex, time, and group (all *p* < 0.001), with medium effect sizes. Significant two- and three-way interactions were observed among sex, time, and group. *Post hoc* analyses confirmed significant reductions in FAT% in the EG for both men and women (*p* < 0.001) and consistent sex differences across conditions (*p* < 0.001).

For LEAN, only sex showed a significant main effect (*F*(1,121) = 19.54, *p* < 0.001, η^2^ = 0.09). The sole significant interaction was sex × group (*F*(1,121) = 7.85, *p* < 0.001, η^2^ = 0.05). No significant pre–post differences were found within groups, but sex differences remained significant across all conditions (*p* < 0.001).

For WAIST, significant main effects were found for sex, time, and group (all *p* < 0.001). Interactions for sex × time, time × group, and sex × time × group were also significant, though sex × group was not. *Post hoc* tests revealed significant reductions from pre- to post-test in the EG for both sexes (*p* < 0.001), along with sex differences in both EG and CG at all time points (*p* < 0.001).

Finally, for waist-to-hip ratio (WHR), significant main effects were found for sex, time, and group (all *p* < 0.001), with small to medium effect sizes. Interactions for sex × time, time × group, and sex × time × group were significant. *Post hoc* results showed that men consistently had higher WHR than women, and the EG showed significant reductions for both sexes (*p* < 0.001), while no changes occurred in the CG.

### 3.2 Cardiovascular outcomes

For BP_SYS, significant main effects were found for time (*F*(1,121) = 13.42, *p* < 0.001, η^2^ = 0.10) and group (*F*(1,121) = 9.11, *p* < 0.001, η^2^ = 0.07), while sex was not significant. Significant sex × group and sex × time × group interactions were observed (*p* ≤ 0.050). *Post hoc* analyses showed significant pre–post reductions in the EG for both women (*p* = 0.010) and men (*p* < 0.001). Sex differences were evident at post-test in both groups (*p* < 0.05), and EG values were significantly lower than CG at post-test (*p* < 0.001 for both sexes).

For BP_DIA, main effects were significant for time (*F*(1,121) = 17.11, *p* < 0.001, η^2^ = 0.12) and group (*F*(1,121) = 10.22, *p* = 0.003, η^2^ = 0.08), with no effect of sex. A time × group interaction was significant (*F*(1,121) = 12.15, *p* < 0.001, η^2^ = 0.09). *Post hoc* tests showed significant pre–post improvements in the EG for both sexes (*p* < 0.001) and lower post-test values in EG vs. CG (*p* < 0.001 for men; *p* = 0.005 for women). No sex differences were observed.

For BPM, significant main effects were found for sex (*F*(1,121) = 20.05, *p* < 0.001, η^2^ = 0.14), time (*F*(1,121) = 4.61, *p* = 0.041, η^2^ = 0.04), and group (*F*(1,121) = 16.48, *p* < 0.001, η^2^ = 0.12). Significant sex × time and time × group interactions were detected (*p* ≤ 0.001). *Post hoc* results revealed significant post-test reductions in the EG for both sexes (men: *p* < 0.001; women: *p* = 0.036), and lower values in EG vs. CG at post-test (*p* < 0.001). No sex differences appeared within CG.

For Double Product (DP), main effects of sex (*F*(1,121) = 14.29, *p* = 0.001, η^2^ = 0.11), time (*F*(1,121) = 13.61, *p* = 0.001, η^2^ = 0.10), and group (*F*(1,121) = 17.98, *p* < 0.001, η^2^ = 0.13) were significant. Sex × time, time × group, and sex × time × group interactions were also significant (*p* ≤ 0.027). *Post hoc* analyses indicated significant reductions in the EG for both men (*p* < 0.001) and women (*p* = 0.050), with no changes in the CG. Sex differences were present in the EG at both pre- and post-test (*p* ≤ 0.028), and EG showed significantly lower post-test DP values compared with CG (*p* < 0.001 for men; *p* = 0.003 for women).

### 3.3 Metabolic outcomes

For the variable GLU, significant main effects were found for sex (*F*(1,121) = 18.25, *p* < 0.001, η^2^ = 0.13), time (*F*(1,121) = 12.44, *p* < 0.001, η^2^ = 0.09), and group (*F*(1,121) = 10.41, *p* < 0.001, η^2^ = 0.08). Significant interactions were observed for sex × time, sex × group, time × group, and sex × time × group (*p* ≤ 0.015). *Post hoc* analyses showed significant sex differences within the EG at pre- and post-test (*p* < 0.001) and pre–post improvements for both men (*p* < 0.001) and women (*p* = 0.002). No significant changes occurred in the CG, while post-test comparisons between EG and CG were significant for both sexes (*p* ≤ 0.013).

For the variable HDL, main effects were significant for sex (*F*(1,121) = 15.93, *p* < 0.001, η^2^ = 0.12), time (*F*(1,121) = 13.11, *p* = 0.001, η^2^ = 0.10), and group (*F*(1,121) = 7.27, *p* < 0.001, η^2^ = 0.06). Sex × time, time × group, and sex × time × group interactions were also significant (*p* ≤ 0.036). *Post hoc* results showed sex differences in both EG and CG at pre- and post-test (*p* ≤ 0.015) and pre–post improvements in the EG (*p* < 0.001), with no changes in the CG. A between-group difference at post-test was found for women (*p* = 0.030), but not for men.

For the variable LDL, main effects were significant for time (*F*(1,121) = 18.43, *p* < 0.001, η^2^ = 0.13) and group (*F*(1,121) = 14.22, *p* < 0.001, η^2^ = 0.11); sex was not significant. Significant interactions were observed for sex × time, sex × group, time × group, and sex × time × group (*p* ≤ 0.011). *Post hoc* analyses showed significant pre–post improvements in the EG for both sexes (*p* < 0.001) and sex differences at post-test within the EG (*p* < 0.001). No changes occurred in the CG, while EG vs. CG post-test differences were significant in both sexes (*p* < 0.001).

For the variable CHO, significant main effects were found for time (*F*(1,121) = 12.35, *p* < 0.001, η^2^ = 0.09) and group (*F*(1,121) = 10.47, *p* < 0.001, η^2^ = 0.08). Significant interactions were observed for sex × time, time × group, and sex × time × group (*p* < 0.001). *Post hoc* analyses showed pre–post reductions in the EG for both sexes (*p* < 0.001) and post-test EG vs. CG differences (*p* < 0.001). In contrast, no group differences were detected at the pre-test for either sex.

For TRYG, main effects were significant for time (*F*(1,121) = 16.32, *p* < 0.001, η^2^ = 0.12), sex (*F*(1,121) = 9.74, *p* < 0.001, η^2^ = 0.08), and group (*F*(1,121) = 13.61, *p* < 0.001, η^2^ = 0.09). Significant interactions were observed for sex × time, time × group, and sex × time × group (*p* < 0.001), while sex × group was not significant. *Post hoc* analyses showed that men in the EG had higher TRYG than women at both pre- and post-test (*p* < 0.001). Pre–post reductions were significant in the EG for both men (*p* < 0.001) and women (*p* = 0.033), with no significant changes in the CG. Between-group differences were present at post-test for both sexes (*p* < 0.001) but not at pre-test.

For the variable URI_AC, main effects were significant for sex (*F*(1,121) = 5.42, *p* < 0.001, η^2^ = 0.04), time (*F*(1,121) = 11.44, *p* < 0.001, η^2^ = 0.09), and group (*F*(1,121) = 9.73, *p* < 0.001, η^2^ = 0.07). Significant interactions were observed for sex × group, time × group, and sex × time × group (*p* ≤ 0.001), while sex × time was not significant. *Post hoc* analyses showed no pre-test differences between EG and CG (*p* ≥ 0.828), but post-test reductions in EG for both sexes (*p* < 0.001). Sex differences persisted within the EG at pre- and post-test (*p* < 0.001), with men having higher values than women.

## 4 Discussion

The present study aimed to evaluate the effects of a combined intervention based on the Mediterranean diet and structured physical exercise—both aligned with World Health Organization recommendations—on anthropometric, cardiovascular, and metabolic variables in a physically inactive but otherwise healthy adult population. The primary finding of this 8-week randomized controlled trial is that the combined Mediterranean diet and structured exercise intervention produced clinically meaningful improvements across multiple physiological domains, with effect sizes ranging from small to large. The experimental group experienced significant improvements in multiple physiological indicators, with consistent differences observed between sexes, over time, and between groups, demonstrating the effectiveness of combined lifestyle interventions that address both dietary and physical activity components simultaneously.

### 4.1 Anthropometric outcomes

The intervention applied to the experimental groups (EGs) resulted in significant improvements in anthropometric variables, including body mass (BM), body composition, and fat distribution in both sexes. The observed effect sizes indicate moderate to large impacts across most variables, reinforcing the effectiveness of the combined Mediterranean diet and exercise protocol. These findings align with previous literature suggesting that the synergy between diet and physical activity produces greater benefits than either strategy alone (23).

The significant reduction in BM in the EG demonstrates a medium effect of the intervention on promoting effective weight loss. The dietary component likely contributed by controlling caloric intake, while the exercise component increased energy expenditure and helped preserve lean mass, acting in a complementary fashion. This is consistent with previous literature reporting that combined interventions yield more pronounced weight loss compared to diet or exercise alone (44, 45). Importantly, such weight loss is strongly linked to a decreased risk of metabolic and cardiovascular diseases (46, 47), highlighting the clinical relevance of the findings.

The decrease in BMI, reflecting not only absolute weight loss but also improvements in body fat distribution, was associated with a moderate to large effect size. Improvements in waist-to-hip ratio (WHR) further emphasize the positive changes in cardiovascular health markers. These findings are aligned with those reported by Esposito et al. (2010) and Martinez-Gonzalez et al. (2019), who documented similar cardiometabolic improvements following Mediterranean diet and exercise interventions (46, 48).

The observed reductions in FAT% represent a moderate to large effect and serve as a key marker of improved body composition. The combination of a healthy diet with regular physical activity likely enhanced fat oxidation and lipolysis, leading to these outcomes. Given the well-established relationship between abdominal adiposity and cardiometabolic risk (49) these reductions have important clinical implications. This magnitude of change is consistent with prior findings from lifestyle interventions incorporating nutrition and exercise (18).

In both men and women of the EG, LEAN showed a slight increase from pre- to post-test, whereas no comparable change was observed in either sex within the CG. Although this improvement did not reach statistical significance and no overall differences emerged between EG and CG, the EG shows a trend toward an increase in lean mass. This pattern suggests that the resistance training component of the intervention may have contributed to the prevention of muscle loss and even to a modest hypertrophic response. Such adaptations are clinically relevant because even small increases in lean mass can positively influence metabolic health, functional performance, and long-term weight regulation, particularly when occurring alongside reductions in total body weight. These results underscore the importance of incorporating structured resistance exercise into dietary interventions, supporting evidence from McCarthy et al. (2021), who emphasized the protective role of combined diet and exercise strategies in maintaining skeletal muscle during weight management programs (50).

Significant reductions in WAIST and WHR, both showing small effect sizes, demonstrate improved fat distribution, particularly in the abdominal area. The presence of three-way interactions (sex × time × group) suggests that the response to intervention on fat distribution varies by sex, potentially reflecting biological differences such as hormonal regulation and fat storage patterns. Since visceral fat is a major risk factor for metabolic diseases, its reduction is highly beneficial. These findings are consistent with those of Klonizakis et al. (2014) and Malakou et al. (2018) who observed meaningful improvements in fat distribution and cardiovascular health following similar combined lifestyle interventions (23, 51).

In summary, the anthropometric results confirm that combining a healthy diet with a physical activity program—including both resistance and cardiovascular training—produces beneficial effects on body composition. These findings are consistent with previous literature reporting synergistic benefits of combined interventions (52). This is evident not only in the individual improvements observed across anthropometric parameters but also in the presence of significant interactions, including three-way interactions (sex × time × group), suggesting a differential and dynamic response to the intervention depending on sex and time. These effects support not only effective weight reduction but also the optimization of body composition and fat distribution—key factors in lowering the risk of metabolic and cardiovascular diseases. Although sex-related differences were detected, likely reflecting biological distinctions such as hormonal influences and fat distribution patterns, these differences did not diminish the effectiveness of the intervention, which proved beneficial for both men and women. Similar findings were reported by Sampaio et al. (2024), who observed significant improvements in body fat and interaction effects following a Mediterranean diet-based multicomponent training program (53).

### 4.2 Cardiovascular outcomes

From an integrative perspective, the findings related to cardiovascular variables confirm the effectiveness of the combined diet and exercise protocol, showing consistent positive effects on BP_SYS, BP_DIA, BPM, and DP—the latter being an indirect indicator of myocardial workload—exclusively in the experimental group (EG) for both men and women, with no significant changes observed in the control group (CG) across the intervention period.

For BP_SYS, the intervention produced significant main effects for both time and group, with effect sizes ranging from small to medium. *Post-hoc* analyses revealed that reductions occurred only in the EG for both sexes, highlighting the direct benefits of adopting a healthy lifestyle combining diet and exercise. These improvements are likely linked to the vasodilatory action of monounsaturated fatty acids and polyphenols in the Mediterranean diet, together with the autonomic and endothelial adaptations induced by regular exercise. De Pergola and D'Alessandro (54) note that polyphenols in olive oil enhance endothelial nitric oxide synthesis and hyperpolarization, facilitating vascular relaxation and lowering BP (54). This is consistent with Esposito et al. (44, 48) and Malakou et al. (23), who showed that the combination of Mediterranean diet and physical activity reduces BP more effectively than either intervention alone. The presence of a three-way interaction (sex × time × group, small effect) indicates that these benefits were modulated by sex, possibly reflecting physiological differences in hemodynamic or hormonal responses. Similar sex-specific patterns in BP regulation have been reported by Pérez-Gimeno et al. (2024), underscoring the importance of stratified analyses (55).

For BP_DIA, the effects were even more robust, with medium effect sizes for time and smaller but significant effects for group. Only the EG exhibited significant reductions in diastolic pressure for both men and women, while the CG showed no meaningful change. This pattern indicates a consistent reduction in peripheral vascular resistance attributable to the intervention. The absence of sex differences suggests that the adaptive mechanisms—such as improved endothelial function or reduced oxidative stress—were similar across sexes within the EG. These results align with findings from the PREDIMED-PLUS study (46) and the PREDIMED trial (56), both of which documented significant diastolic BP reductions following Mediterranean diet interventions, with potential long-term implications for reducing stroke and cardiovascular risk. The meta-analysis by Bakaloudi et al. (57) further supports the association between higher adherence to the Mediterranean diet and lower systolic BP. However, diastolic effects were less consistent in observational studies.

In BPM, significant main effects for sex, time, and group were observed, with effect sizes ranging from small to medium, and notable sex × time and time × group interactions. The EG showed significant decreases in resting heart rate for both men and women, whereas the CG maintained stable values. These reductions suggest enhanced vagal tone and cardiovascular efficiency, consistent with adaptations to regular aerobic exercise, and may have been reinforced by reductions in body weight and improved metabolic control. The observed sex-related differences, although modest, may point to slightly greater absolute decreases among men. This aligns with Tuttolomondo et al. (58), who reported that the Mediterranean diet exerts anti-inflammatory and metabolic effects, including reductions in resting heart rate.

Finally, for DP, significant main effects for sex, time, and group were detected, along with sex × time, time × group, and three-way interactions, with small-to-medium effect sizes. Significant reductions were observed only in the EG for both sexes, with no change in the CG. These decreases reflect a clinically meaningful reduction in myocardial oxygen demand, indicating lower cardiac workload at rest and potentially reduced ischemic risk. This outcome likely resulted from the combined effect of lower BP and heart rate. The three-way interaction suggests that hemodynamic adaptations differed slightly between men and women, though both benefited substantially within the EG. Klonizakis et al. (51) reported similar improvements in endothelial function following combined diet–exercise interventions, even without marked weight loss (51), while López-Gil et al. (59) found comparable cardiometabolic benefits in younger populations, suggesting that these effects may be generalizable across age groups (59).

Overall, these results highlight the high efficacy of the intervention in improving cardiovascular outcomes in physically inactive adults, with benefits consistently confined to the EG. The observed changes appear to derive from the synergistic vasoprotective, antioxidant, and anti-inflammatory effects of the Mediterranean diet combined with the cardiorregulatory adaptations from structured exercise. As noted by Tuttolomondo et al. (58), such synergy may act at molecular and systemic levels, involving epigenetic regulation, modulation of gut microbiota, and improved endothelial function. The magnitude and consistency of these improvements—together with significant and clinically relevant interactions—underscore the value of implementing tailored combined strategies as part of comprehensive cardiovascular prevention programs.

### 4.3 Metabolic outcomes

Regarding the metabolic variables assessed in this study, the findings provide support for the effectiveness of the combined diet and physical exercise intervention, demonstrating significant improvements in key indicators of metabolic health. These improvements are fully consistent with previous research (60), which highlights the beneficial role of multicomponent interventions in optimizing parameters such as glucose, lipid profile, and uric acid, even in adults without diagnosed metabolic disorders but with physically inactive lifestyles.

GLU decreased significantly in the experimental group (EG) for both sexes, with a small effect size for time and a small effect for group, indicating that improvements occurred during the intervention period and were more pronounced among those receiving the combined program. This pattern is consistent with findings from studies such as those by Esposito et al. (44, 48) and Babio et al. (47), which reported significant improvements in glycemic regulation following combined Mediterranean diet and exercise interventions. Additionally, Celik and Yildiz (2021) observed reductions in glucose and HbA1c associated with a Mediterranean diet and physical training program (18). Similarly, Gorini et al. (2025) (61) found that fasting glucose levels significantly decreased in both men and women following a Mediterranean diet and physical activity intervention, highlighting the broad glycemic benefits of such programs. These effects are further supported by meta-analytic evidence, as reported by Silva et al. (62), who found significant reductions in fasting glucose, insulin, and HOMA-IR in physically inactive adults after combined aerobic and resistance training. While Zhao et al. (2021) demonstrated similar improvements in populations with obesity and type 2 diabetes (63), these findings help contextualize the potential of such interventions to prevent metabolic dysregulation even in non-clinical populations. Although the reductions in GLU observed in our study were statistically significant and accompanied by small effect sizes, it is important to note that all values remained within the normal glycemic range at both baseline and post-intervention. Therefore, the clinical relevance of these changes may be limited in metabolically healthy individuals. Nonetheless, these improvements may reflect enhanced metabolic efficiency or early preventive benefits, particularly in populations at risk for future metabolic disorders. Although sex-related differences were statistically significant, they do not compromise the validity of the intervention's effects and likely reflect physiological disparities in insulin sensitivity and fat distribution between sexes, with men being more prone to visceral fat accumulation, potentially explaining their more pronounced glycemic response.

With regard to HDL, the intervention led to significant improvements, particularly in women, which is clinically relevant due to its protective role against cardiovascular disease. The effect size for group was small, indicating a meaningful yet modest magnitude of change attributable to the combined program. This increase may be linked to greater lipid metabolism activation induced by exercise in women, possibly related to hormonal factors and lower visceral fat mass. These findings align with those reported by Martínez-González et al. (46) and McCarthy and Berg (50), who also observed HDL increases following interventions combining a Mediterranean diet and physical activity. Gorini et al. (61) similarly documented a significant rise in HDL cholesterol among women, reinforcing the idea that sex-specific physiological mechanisms may enhance lipid profile responses to lifestyle interventions (61).

In terms of LDL, significant reductions were observed following the intervention, with particularly favorable effects in women, although benefits were also evident in men. The reductions showed medium effect sizes for both time and group, reflecting modest but clinically relevant improvements. This difference may be due to a stronger effect of the Mediterranean diet on lipid profiles in women, possibly related to greater adherence and metabolic responsiveness to healthy dietary patterns. This trend has been previously documented by Hermsdorff et al. (45), who emphasize the impact of combined interventions on lowering LDL and improving overall lipid profiles. In line with these findings, Gorini et al. (61) reported more pronounced reductions in LDL among men, along with increases in HDL in women, underscoring the importance of sex-specific responses when designing lifestyle interventions (61).

CHO also showed a meaningful reduction after the intervention, with a medium effect size for time and medium for group, consistent with prior evidence linking the Mediterranean diet and regular exercise with sustained decreases in this marker. The magnitude of the change suggests a global improvement in lipid metabolism, resulting from the combined effect of reduced saturated fat intake and increased physical activity. Similar outcomes were reported by Esposito et al. (48) and Malakou et al. (23), who emphasized the efficacy of the combined approach in improving lipid profiles, regardless of sex-related differences observed at the end of the protocol. Gorini et al. (61) also found significant reductions in total cholesterol, particularly in men, supporting the view that combined diet and physical activity interventions effectively enhance lipid profiles across sexes (61).

TRYG were also significantly reduced in the experimental group, with a medium effect size for time and group, indicating a stronger temporal effect of the intervention. A positive metabolic response was observed in both sexes, consistent with findings by Di Renzo et al. (64), who demonstrated that personalized Mediterranean diet combined with physical activity therapy significantly improved lipid profiles and reduced cardiovascular risk indexes, including reductions in atherogenic markers linked to both weight and fat mass decrease. The decrease in triglycerides may be mediated by enhanced fatty acid oxidation induced by exercise, along with lower availability of refined carbohydrates.

For URI_AC, the intervention led to significant reductions, with a small effect size for time, which is particularly relevant given its association with purine metabolism and inflammatory processes. Research has shown that specific dietary patterns can reduce uric acid levels, as found by Martínez-González et al. (46) and Babio et al. (47), which support the effect of the Mediterranean diet combined with exercise in improving the overall metabolic profile, including this biomarker. The reduction in uric acid may reflect lower endogenous production resulting from decreased chronic inflammation and oxidative stress, as well as potential changes in dietary protein patterns.

In summary, the results observed across all metabolic variables indicate that the combined intervention was highly effective in optimizing glycemic control, lipid profiles, and uric acid metabolism, regardless of sex. However, quantitative differences between men and women were evident and likely attributable to biological factors. Across these parameters, the magnitude of change ranged from small to medium effect sizes, supporting both the statistical and clinical relevance of the observed benefits. These improvements are consistent with the findings of previous studies, particularly the PREDIMED trial (25), which analyzed multiple metabolic parameters under combined interventions and demonstrated similar small to medium effect sizes in cardiovascular and metabolic outcomes. The results support the effectiveness of combining the Mediterranean diet with structured physical activity. This aligns with evidence from previous studies emphasizing the synergistic benefits of multicomponent interventions over isolated dietary or exercise approaches (18, 48, 50). While the population in the present study was healthy but physically inactive, the findings support the potential of such protocols in the prevention of metabolic disorders commonly associated with metabolic syndrome, type 2 diabetes, and cardiovascular disease. These findings underscore the need to implement comprehensive and personalized intervention strategies in clinical and community settings.

Despite the positive results, this study presents certain limitations that should be considered when interpreting the findings and may inform future research. First, the absence of isolated intervention groups (diet only or exercise only) prevented the assessment of the independent effects of each component. Including these groups would have allowed for a clearer understanding of the individual contributions of the Mediterranean diet and physical activity to the observed improvements in metabolic, anthropometric, and cardiovascular outcomes. Second, while the 8-week intervention detected clinically meaningful changes, this duration may be insufficient to assess long-term sustainability of effects, adherence maintenance, or potential metabolic rebound. Extended follow-up periods are needed to evaluate the durability of these cardiometabolic improvements. Third, the homogeneity of the sample—a relatively uniform demographic group—may limit the generalizability of the findings to other populations with different characteristics or comorbid conditions. Finally, although a wide range of variables was assessed, key inflammatory and hormonal markers such as C-reactive protein, leptin, adiponectin, or insulin were not included. These biomarkers could have provided a deeper characterization of the participants' metabolic status and the physiological impact of the intervention.

## 5 Conclusion

The present study provides robust evidence that a lifestyle intervention combining the Mediterranean diet with structured physical exercise is an effective and feasible strategy for improving cardiometabolic health in physically inactive adults. This culturally adapted approach in a non-Mediterranean population, combined with comprehensive sex-stratified analysis, addresses key gaps in preventive health research. Through an integrative, non-pharmacological approach targeting multiple health domains, the intervention aligns with global prevention guidelines. It demonstrates clear potential for application in both clinical and public health contexts, particularly for preventing metabolic syndrome in physically inactive adults aged 35–50.

The strength of this intervention lies in its multicomponent design, which targets key lifestyle factors simultaneously and promotes sustainable behavioral changes. Its effectiveness across sexes further supports its broad applicability, although sex-based differences observed throughout the study highlight the need for tailored strategies in future implementations.

While the absence of isolated intervention arms and the relative homogeneity of the sample represent methodological limitations, the consistency of the outcomes reinforces the clinical relevance of this approach. Future research should build on these findings by examining the long-term sustainability of the effects, exploring additional biomarkers, and expanding the intervention to more diverse populations and settings.

## Data Availability

The raw data supporting the conclusions of this article will be made available by the authors, without undue reservation.
